# A Mobile Application for Enhancing Caregiver Support and Resource Management for Long-Term Dependent Individuals in Rural Areas

**DOI:** 10.3390/healthcare12151473

**Published:** 2024-07-24

**Authors:** Niruwan Turnbull, Chanaphol Sriruecha, Ruchakron Kongmant, Le Ke Nghiep, Kukiat Tudpor

**Affiliations:** 1 Faculty of Public Health and Public Health and Environmental Policy in Southeast Asia Research Cluster (PHEP-SEA), Mahasarakham University, Maha Sarakham 44150, Thailand; niruwan.o@msu.ac.th; 2 Faculty of Science and Technology, College of Asian Scholars, Khon Kaen 40000, Thailand; chanaphol@cas.ac.th; 3 Public Health and Environmental Policy in Southeast Asia Research Cluster (PHEP-SEA), Mahasarakham University, Maha Sarakham 44150, Thailand; ruchaorn.kon@kkumail.com; 4 Vinh Long Department of Health, Vinh Long 85000, Vietnam; lekenghiep@gmail.com

**Keywords:** mobile application, caregivers, long-term dependent individuals, geographic information system, technology acceptance model

## Abstract

The “*SmartCaregivers*” 1.0 mobile application is a beacon of hope for caregivers (CG) in rural areas, often facing limited access to facilities and support. This study, conducted from February to August 2021, aimed to comprehensively analyze the need for developing a database system and a mobile application tailored to enhance caregiver support and resource management for long-term dependent individuals in the rural areas of Maha Sarakham province, Thailand. The research followed a rigorous research and development (R & D) approach, specifically the ADDIE model (analysis, design, development, implementation, and evaluation). Data were collected from 402 caregivers and 10 key informants through surveys and interviews, as well as from 402 caregivers during the implementation and evaluation phases. The application’s impact was assessed using a quasi-experimental design with a one-group pre–post-test, and its acceptance was evaluated through the technology acceptance model (TAM). The application significantly improved caregivers’ knowledge scores, with a mean increase from 10.49 ± 2.53 to 12.18 ± 2.76 post-intervention. High scores for perceived usefulness (4.36 ± 0.62) and ease of use (4.31 ± 0.59) reassure the audience about the application’s effectiveness in providing rapid access to health information, aiding decision-making, and improving care coordination. The system quality was also highly rated, with users appreciating the variety of functions and structural design. This potential for transformation and improvement instills hope and optimism for the future of caregiving in rural areas.

## 1. Introduction

Globally, the population of older individuals is increasing rapidly. According to the World Health Organization (WHO), the number of people aged 60 years and older is expected to rise from 900 million in 2015 to 2 billion by 2050, representing 22% of the world’s population [1]. According to the most recent data from a survey conducted in 2021, the number of older adults has significantly increased to 19% of all people [2]. This demographic shift substantially affects healthcare systems and caregiving practices [3]. Caregivers are essential to the health and well-being of older and dependent individuals by assisting with daily care activities, medical assistance, and emotional support to improve their quality of life [4].

Caregivers in rural Thailand face unique challenges due to the geographical and socio-economic landscape [5]. The region’s rural setting often limits access to healthcare workforce, facilities, and support, exacerbating the difficulties experienced by caregivers [6]. According to Anderson and Thompson, rural caregivers are more likely to experience higher stress levels and burnout due to the lack of accessible resources and support networks [7,8]. This information highlights the critical need for tailored interventions that cater to the specific needs of caregivers in rural areas.

A new era of technology-enabled interventions that can empower and support caregivers has emerged, thanks to new technologies like mobile applications, robotics, connected sensors, virtual/augmented/mixed reality, and ever-ubiquitous tools backed by advanced data analytics [9]. Previous studies have demonstrated the effectiveness of mobile health applications in supporting caregivers. For instance, developing a mobile application for caregivers of dementia patients showed significant improvements in caregiver well-being and patient management [10]. Similarly, a study by Kelly and colleagues highlighted the positive impact of mobile applications on caregiver stress reduction and care coordination [11]. These findings underscore the potential benefits of mobile health interventions in various caregiving contexts. This study, conducted from February to August 2021, aimed to comprehensively analyze the need for developing a database system and a mobile application tailored to enhance caregiver support and resource management for long-term dependent individuals in the rural areas of Maha Sarakham province, Thailand. We hypothesized that the application could provide tools for managing care routines, accessing local resources, and improving communication with healthcare providers. By leveraging the capabilities of mobile technology, the application seeks to enhance the quality of care provided to dependent individuals and reduce the burden on caregivers.

## 2. Materials and Methods

### 2.1. Research Design and Setting

**Figure 1:**
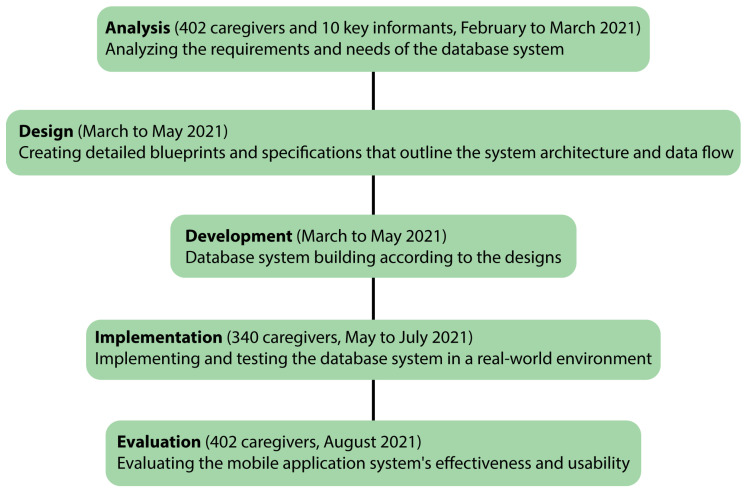
ADDIE model, participants, and timeframe.

The research employed a research and development (R & D) method to develop a mobile application for caregivers of long-term dependent individuals in Maha Sarakham province, utilizing the ADDIE model (analysis, design, development, implementation, and evaluation) model. The ADDIE model is a systematic instructional design framework with five phases (Figure 1).

In the evaluation phase, we employed the technology acceptance model (TAM) to test caregivers’ perception of the mobile application. TAM assesses two main factors: perceived usefulness and perceived ease of use. Caregivers were surveyed to determine how these factors influenced their acceptance and use of the application. Feedback collected through TAM helped identify areas for improvement and gauge overall satisfaction. This phase also includes refining the database system based on user feedback and developing it into a fully functional smartphone application to enhance accessibility and usability.

### 2.2. Participants and Study Areas

The participants were separated into two groups: 10 key informants, who participated in the analysis, design, and development phases; and 402 caregivers, who participated in the analysis, design, development, implementation, and evaluation phases. The ten key informants included administrators, project managers from local government organizations, staff from subdistrict health-promoting hospitals, long-term care managers, representatives of village health volunteers (VHVs), caregivers, relatives, and members of community senior clubs. Their insights and expertise were crucial in shaping the mobile application to meet caregivers’ needs effectively. They provided valuable information on the requirements and challenges faced by caregivers, contributed to the design of the user interface, and helped develop features that address specific needs. Caregivers in the implementation and evaluation phases were selected using cluster sampling from 32 target areas. Only caregivers who had undergone capacity development by a recognized institution and volunteered to participate were included.

**Figure 2:**
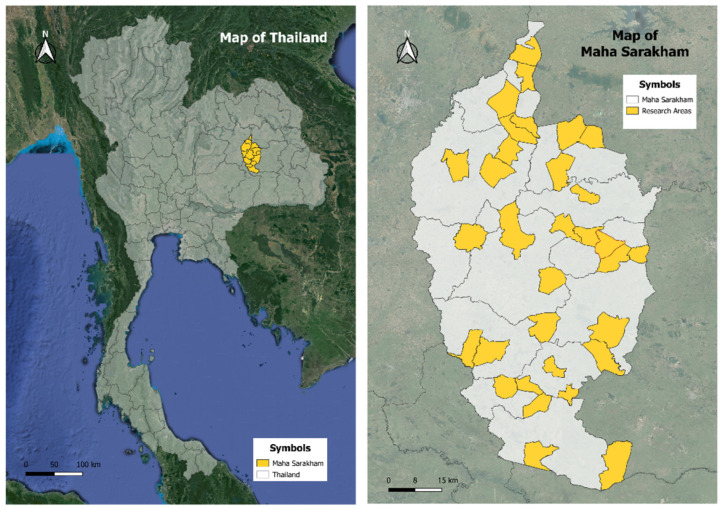
The research areas of the participants.

The study was conducted in Maha Sarakham province, located in the Northeastern region of Thailand. Maha Sarakham province is divided into 13 districts: Borabue, Chiang Yuen, Chuen Chom, Kae Dam, Kantharawichai, Kosum Phisai, Kut Rang, Mueang Maha Sarakham, Na Chueak, Na Dun, Phayakkhaphum Phisai, Wapi Pathum, and Yang Sisurat. Each district has unique demographic and geographic characteristics, predominantly rural areas. Known for their solid agricultural activities, local traditions, and community networks, these districts provide a comprehensive backdrop for the study. The geographic information system (GIS) program was used to map the participant locations. The global positioning system (GPS) was used to gather participant data. The World Geodetic System 1984, Universal Transverse Mercator coordinate system (EPSG:32648 WGS 84/UTM Zone 48N) was the geographic coordinate system utilized in this investigation. By including all districts, the study ensured a diverse range of environments is represented, capturing comprehensive and representative findings across Maha Sarakham province (Figure 2).

### 2.3. Data Collection

The data collection process for this study was meticulously designed and executed in three sections, following the ADDIE model. The study was conducted from February to August 2021. This comprehensive approach ensured systematic and thorough data gathering to support the development and evaluation of the mobile application for caregivers. Questionnaires are available in English (Supplementary Data S1) and Thai (Supplementary Data S2).

#### 2.3.1. Phase 1: Analysis

In the analysis phase, the primary focus was on understanding the requirements and need for developing a database system for the mobile application. As part of a comprehensive needs assessment, data were collected through surveys and interviews. This assessment aimed to gather qualitative and quantitative information about the challenges caregivers face, their specific needs, and their expectations for a mobile app to address them. A thorough literature review on caregiver support, mobile health applications, and associated technologies was conducted to identify gaps and guide the development process. Qualitative data from in-depth interviews were summarized from five critical questions: What information do caregivers need regarding long-term care? How can knowledge management be improved? What are the mapping and tracking needs for caregivers? How can reporting be streamlined? What are the biggest problems in caring for people with dependence? These questions helped capture the essence of caregivers’ experiences and informed the design and development of the mobile application.

#### 2.3.2. Phases 2 and 3: Design and Development

In the design and development phases, we gathered ten key informants to focus on creating and building the database system and mobile application. Data collection activities included user interface (UI) design workshops (collaborative sessions with caregivers and stakeholders) to design intuitive and user-friendly interfaces for the application. Prototyping and user experience questionnaires (UEQ) gathered feedback on the design, functionality, and overall user experience. The iterative feedback loop continuously collected user feedback during development to refine and improve the application, ensuring it meets user expectations and requirements. The source code for the application is publicly available [12].

#### 2.3.3. Phases 4 and 5: Implementation and Evaluation

In these phases, the application effectiveness was evaluated. The data collection activities included a quasi-experiment using a one-group pre–post-test design to assess its impact on caregivers using the mobile application. TAM was used to evaluate 340 caregivers from 32 local health promotion hospitals around Maha Sarakham province using the mobile application over three months. Two main factors were perceived usefulness and ease of use [13]. The assessment provided insights into how likely caregivers would be to adopt and integrate the application into their daily routines based on their perceptions of its usefulness and ease of use.

### 2.4. Data Analysis

The research utilized both descriptive and inferential statistics to analyze the quantitative data. Descriptive statistics, including average (mean), minimum (min), maximum (max), standard deviation (SD), and percentage, were employed to summarize the demographic characteristics and baseline measures. Inferential statistics were conducted using a paired *t*-test to compare pre- and post-intervention scores. This analysis has been applied to demonstrate the effectiveness of the intervention by identifying significant changes in key measures. The combined use of descriptive and inferential statistics provided a comprehensive understanding of the data and the impact of the intervention. For qualitative data, we summarized information from group discussion and brainstorming based on the Grounded Theory [14].

### 2.5. Ethical Considerations

The study was conducted by the principles of human research ethics. Ethical approval was obtained from the Human Research Ethics Committee of the Maha Sarakham Provincial Public Health Office, with approval number 2/2564, dated 1 February 2021.

## 3. Results

Three sections reported the results: analysis, design and development, and implementation and evaluation. A total of 457 caregivers met the criteria for inclusion in the study, and 402 consented to participate in the research, representing 88.00% of the eligible participants. These caregivers tested the application and provided feedback on its usability, effectiveness, and impact on their caregiving routines. These results involved 412 participants, including 402 caregivers and ten stakeholders.

### 3.1. Analysis Phase of Survey and Interviews of Needs Assessment Findings

The purposive sampling led to the selection of 10 stakeholders (five healthcare professionals and five public administrators). The selected professionals included four care managers from hospitals or health promotion hospitals: Kosum Phisai Hospital, Kae Dam Hospital, Nong Saeng Health Promotion Hospital, and Chuen Chom Health Promotion Hospital, along with the Director of Non Piban Health Promotion Hospital. Additionally, representatives from local government organizations comprised the Head of the Health and Environmental Division from Maha Sarakham Municipality, two care managers from local administrative organizations (Kae Dam Subdistrict Municipality and Chiang Yuen Subdistrict Municipality), and two Presidents of Subdistrict Administrative Organizations/Municipalities (Wang Saeng Subdistrict Administrative Organization and Na Chueak Subdistrict Municipality).

The socio-economic data showed the 402 participants predominantly consisted of females (95.8%) with an average age of 49.89 years (SD = 8.14, Max = 75 years, Min = 23 years), most of whom were in the middle age group (83.6%). The majority were married (78.6%), and households typically had 1–4 members (47.8%) or 5–7 members (48.0%), with an average of 4.71 people per household (SD = 1.60, Max = 10, Min = 1). Most participants lived with their spouse (67.9%) or with children/grandchildren (19.4%). Regarding education, a significant portion had secondary education (73.1%). The average monthly income was 4193.88 Baht (approximately 128 USD), SD = 3350.77, Max = 40,000 Baht (1227 USD), Min = 600 Baht (18 USD), with most earning less than 5000 Baht (approximately 153 USD), 59.5%. Income sufficiency was reported as barely sufficient (53.2%) or insufficient and in debt (38.6%). A high need for developing a database system for the mobile application was expressed by the majority (60.26%) or the highest need (35.15%), with a mean score of 4.11 (SD = 0.69, Min = 2.96, Max = 5.00) (See Table 1 and Supplementary Data S3).

The in-depth interview results showed that the analysis phase revealed several critical issues related to dependent individuals’ long-term care (LTC). Caregivers expressed a need for comprehensive information on LTC, including knowledge management via mobile applications like Line for sharing vital knowledge, managing urgent symptoms, adapting to changing patient groups, and maintaining transparent data collection formats. They also highlighted the necessity for unified monthly responsibility zones and efficient check-in and check-out processes using the Thai Chana application. Financial management and reporting were significant concerns, with suggestions for monthly application submissions to reduce paperwork, detailed expense tracking, maintaining home visits and CG performance records, excessive documentation, system redundancies, and offline functionality. Caregivers emphasized the importance of a clear format, linking care plans, and addressing internet recharge issues. Training needs were identified for CGs with limited application proficiency, and additional courses were taken to alleviate the workload of care managers (CMs). Finally, the principal problems in caring for dependent individuals included insufficient patient information and addresses, inadequate equipment due to budget constraints, frequent changes in patient groups, and the need for comprehensive patient data and budget tracking per case.

In addition to in-depth interviews regarding the need for the mobile application, some caregivers suggested: “Currently, we document older health information on paper to submit reports to healthcare professionals, which is evidence of our work. Utilizing a mobile application for data collection would significantly enhance our efficiency and accuracy” (Caregiver A). Another healthcare professional commented: “Our routine work in the local hospital exceeds our duties. Gathering data from caregivers who submit paper reports consumes much of my working time” (Healthcare Professional B) (Supplementary Data S4).

### 3.2. Application Design and Development

**Figure 3:**
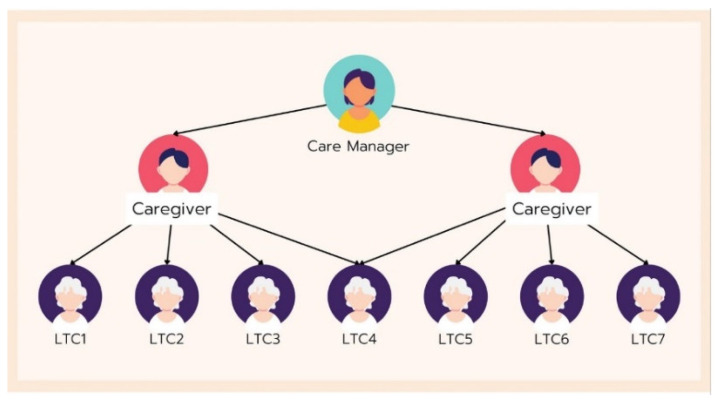
Patient responsibility chart by caregivers.

**Figure 4:**
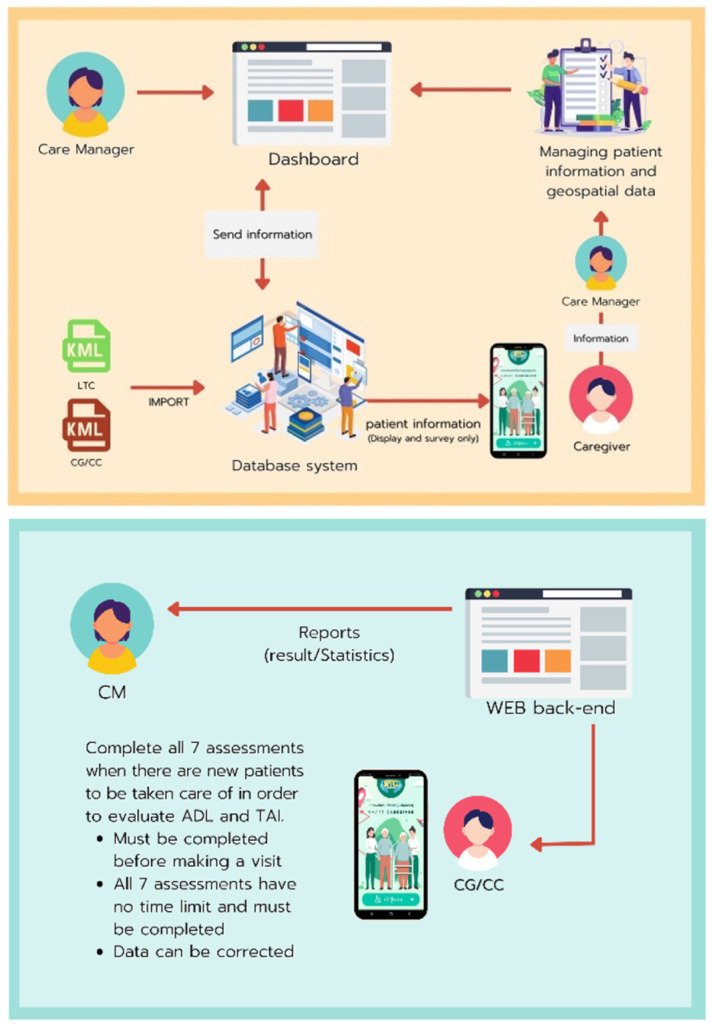
Flowchart of the data retrieval in the system. ADL, activities of daily living, refers to daily activities that people do without assistance. These activities include eating, bathing, dressing, toileting, transferring (walking), and continence. In the context of our study, ADL scores help assess the functional status and independence level of the dependent individuals. TAI, typology of the aged with illustration, is a tool for measuring the ability to do activities (function) of older adults by measuring four functions: (1) movement (motility); (2) mental and intellectual aspects (mental); (3) eating aspect; and (4) toilet.

**Figure 5:**
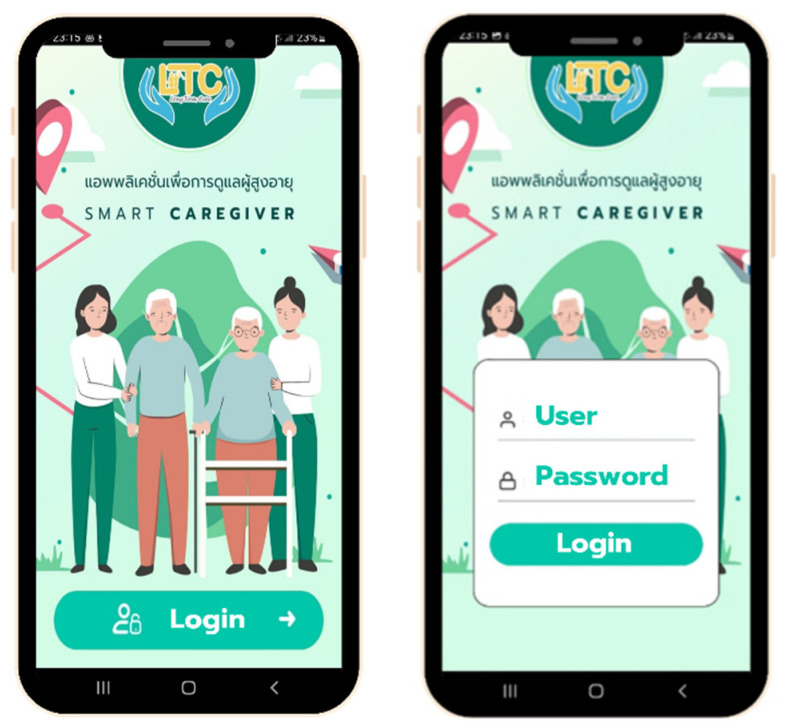
Homepage and login page. The screen shows LTC (long-term care), and Thai texts read “Application for senior care”.

**Figure 6:**
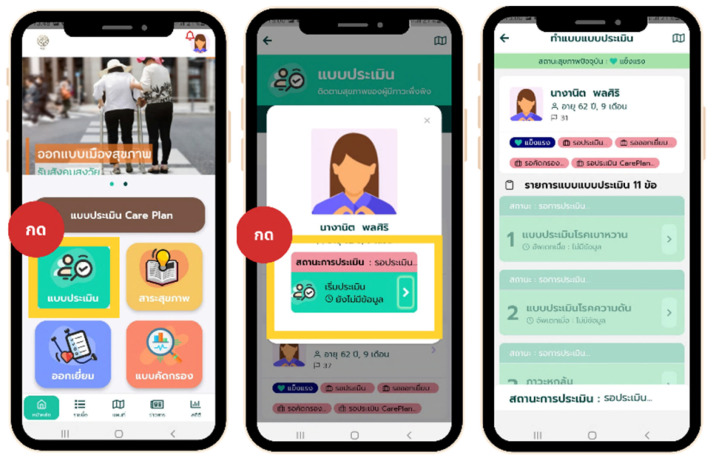
Illustration of health assessment menus in the “*SmartCaregivers*” mobile application. On the left panel, orange and green texts read “Healthy city design” and “Older society support”. The red oval and brown rectangle texts show the “Press” and Care plan assessment forms. The four tabs below show the health assessment form (green), health information (orange), home visit (blue), and screening tools (red). These menus are designed to facilitate comprehensive health assessments by caregivers and include the following components: (1) Health Status Overview: Provides a summary of the overall health status of the dependent individual, including vital signs and general health indicators; (2) ADL (Activities of Daily Living) Assessment: Evaluates the individual’s ability to perform daily activities independently, such as eating, bathing, dressing, toileting, transferring, and continence; (3) TAI (Thai ADL Index) Assessment: This assessment uses a standardized tool tailored to the Thai population to measure the individual’s functional status and independence level in daily activities; (4) Cognitive Function Assessment: Includes tests and evaluations to assess the dependent individual’s cognitive abilities and mental health status; (5) Nutritional Assessment: Monitors dietary intake and nutritional status, ensuring that the individual receives adequate nutrition; (6) Medication Management: Tracks medication schedules, dosages, and adherence to prescribed treatments; and (7) Health Records: Maintains a comprehensive record of all health assessments, medical history, and treatment plans for easy access and reference by caregivers and healthcare professionals. These health assessment menus are integrated into the application to provide caregivers with a robust tool for managing and monitoring the health of dependent individuals effectively. The middle panel, inside the health assessment form (green) menu, below the profile picture, will show the name of the older adult under the supervision of the CG. The status of the assessment is shown in red. The green button can be clicked to start the assessment. On the right panel, the top of the screen indicates that the assessment is in progress. The green bar shows the client’s health status (healthy, etc.). Next to the profile picture, name, last name, age, and number are displayed. Below are the health statuses (healthy, waiting for assessment, home visits, screening, and care planning). Next are 11 screening tools of choice (1, diabetic screening tool; 2, hypertension screening tool; and 3, fall risk screening tool).

The mobile application development involved several vital components and features, as illustrated in Figure 3, Figure 4, Figure 5 and Figure 6. The patient responsibility chart for caregivers (Figure 3) delineates the roles and responsibilities of caregivers, improving organization and confidence in their duties. The flowchart of the data retrieval in the system (Figure 4) outlines an intuitive and efficient process for extracting and managing patient data, ensuring quick and accurate access for timely decision-making. The design of the homepage and login page (Figure 5) emphasizes user-friendly navigation and security, providing caregivers with easy access to essential features while protecting patient data. The health assessment menus (Figure 6) include tools for conducting comprehensive health assessments, allowing caregivers to record and monitor health metrics efficiently.

### 3.3. Application Implementation and Evaluation

Three hundred forty caregivers from 32 local health promotion hospitals around Maha Sarakham province used the mobile application over three months. These caregivers were involved in testing the application and providing feedback on its usability, effectiveness, and impact on their caregiving routines. The quasi-experimental design was implemented using a one-group pre-post-test to assess the effects of the “*SmartCaregivers*” mobile application on caregivers. A total of 340 caregivers participated in the study. The results indicate a significant improvement in the caregivers’ knowledge scores after using the “*SmartCaregivers*” mobile application. The mean knowledge score increased from 10.49 (SD = 2.53) before using the application to 12.18 (SD = 2.76) after using it. The mean difference was −1.69, with a 95% confidence interval ranging from −2.15 to −1.22. The t-value was −7.12, with a df of 339 and a *p*-value of <0.001, indicating that the improvement in knowledge scores was statistically significant. (Table 2).

The application system was then evaluated by applying the TAM and divided into six components: perceived usefulness; perceived ease of use; attitude toward using the application; behavioral intention to use the application; actual system use; and system quality. Data were collected from 340 caregivers within the Maha Sarakham province network area. The majority of users found the “*SmartCaregivers*” mobile application highly beneficial for providing rapid access to health information and updates (mean = 4.36, SD = 0.62), aiding in receiving accurate and reliable health information (mean = 4.30, SD = 0.53), and beneficial for making decisions about caring for dependent individuals in the community (mean = 4.30, SD = 0.57). Moreover, the application was perceived as helpful in facilitating coordination between internal and external organizations (mean = 4.26, SD = 0.60) (Table 3).

The evaluation of the perceived ease of use indicated that most users found the “*SmartCaregivers*” mobile application to be highly convenient and efficient for caregiving tasks for dependent individuals (mean = 4.31, SD = 0.59). Users also reported that the application was easily accessible and convenient to use on mobile devices (mean = 4.26, SD = 0.60) and improved the accuracy of analyzing the locations of dependent individuals (Mean = 4.18, SD = 0.60). However, the less favorable feedback aspect was that the application reduced the steps required for tasks (mean = 4.11, SD = 0.61) (Table 3). Users’ attitudes and interest in using the “*SmartCaregivers*” mobile application assessment revealed that most users believed the application could be practically implemented in their work (mean = 4.24, SD = 0.57). Additionally, users felt the application was important for integration with the health service system (mean = 4.19, SD = 0.57) and expressed a willingness to use the application in their current tasks (mean = 4.13, SD = 0.58). The area with the slightest interest was using the application to develop other work areas (mean = 4.03, SD = 0.64). (Table 3).

Behavioral intention to use the “*SmartCaregivers*” mobile application evaluation showed that most users intended to use the application to assist in planning operations to improve caregiving for dependent individuals (mean = 4.19, SD = 0.61). Additionally, users firmly intended to use the application to manage the database and develop the health service system for dependent individuals (mean = 4.18, SD = 0.60). They also intended to use the application for planning operations to enhance further caregiving (mean = 4.17, SD = 0.61). The aspect with the least favorable response was the perception that using the application would increase their workload (mean = 3.36, SD = 1.08) (Table 3). The evaluation of actual system use revealed that most users were confident using the application to assist in caregiving operations for dependent individuals (mean = 4.11, SD = 0.61). Users also applied the application to analyze health situations for planning care (mean = 3.89, SD = 0.67) and to forecast future health trends of dependent individuals (mean = 3.88, SD = 0.71). The aspect with the least favorable feedback was users’ knowledge of using various menus within the application (Mean = 3.66, SD = 0.74) (Table 3). Lastly, the system quality evaluation indicated that most users believed the application had various functions (mean = 4.02, SD = 0.59). Users also thought the application had an excellent structural system (mean = 4.00, SD = 0.58) and appropriate functions (Mean = 3.93, SD = 0.61). The aspect with the least favorable feedback was the perceived ease of use (mean = 3.85, SD = 0.65) (Table 3).

## 4. Discussion

This study demonstrates the effectiveness of the “*SmartCaregivers*” mobile application in enhancing caregiving practices for long-term dependent individuals in rural areas of Maha Sarakham province, Thailand. The significant improvement in caregivers’ knowledge scores post-intervention highlights the application’s role in providing critical health information and decision-support tools. The high mean scores for perceived usefulness and ease of use indicate that the application successfully addressed the primary needs of caregivers, such as quick access to accurate health information, efficient care management, and improved communication with healthcare providers.

The application’s ability to facilitate the coordination of care activities and provide reliable health data has been particularly beneficial for caregivers operating in rural settings with limited resources. This scenario aligns with findings from other studies that emphasize the importance of technology in improving caregiver efficiency and reducing stress [15]. Mobile applications tailored for caregivers have been shown to enhance care coordination, provide real-time support, and offer educational resources, collectively improving caregiver well-being and patient outcomes [10]. A study by Amiri and colleagues demonstrated that a mobile application designed for Alzheimer’s caregivers significantly improved caregiver well-being and patient management by providing timely information and support [16]. Similarly, Lee and co-workers highlighted the positive impact of mobile health applications on better communication between caregivers and community-dwelling older adults [17]. Recently, another study by Garavand and colleagues found that mobile applications improved the accuracy and efficiency of health data management, which is essential for making informed care decisions [18].

Moreover, the iterative feedback loop involving caregivers in the design and development process ensured the application was user-friendly and met their specific requirements. This approach is consistent with the principles of user-centered design, which has been shown to enhance the adoption and effectiveness of health technologies [19,20]. The positive reception of the “*SmartCaregivers*” application, as reflected in the high scores for system quality and user satisfaction, underscores the importance of engaging end-users in the development process to create practical and effective solutions. Additionally, the study’s findings highlight the potential of mobile health applications to address the challenges caregivers face in rural areas. The application’s ability to provide quick access to health information, facilitate care coordination, and support decision-making processes has been particularly beneficial in settings where access to healthcare resources is limited. This usefulness is supported by research indicating that telemedicine can bridge the gap in healthcare access and support for caregivers in underserved regions [21].

Compared to existing mobile health applications for caregivers, the “*SmartCaregivers*” application offers unique contributions explicitly tailored to rural settings like Maha Sarakham province. The findings of this study have several practical implications for caregivers, healthcare providers, and policymakers. The application has the potential to provide a comprehensive tool for caregivers that enhances caregiving efficiency, reduces the physical and emotional burden, and improves overall care quality for dependent individuals. While our study demonstrated significant improvements in caregiver knowledge, further research is needed to fully evaluate its impact on caregiving outcomes and the well-being of dependent individuals. Healthcare providers can leverage the application to streamline communication and coordination with caregivers, ensuring timely and accurate health interventions. Policymakers can consider integrating such digital health solutions into broader healthcare strategies to address the challenges faced by caregivers, particularly in rural and underserved areas [22].

This study has three main limitations. First, the quasi-experimental design with a one-group pre–post-test used in our study limits its internal validity and the ability to establish causality. While this design provided initial evidence of the app’s effectiveness, future research should incorporate a randomized controlled trial (RCT) to establish causal relationships better. An RCT would allow for comparing intervention and control groups, providing more robust evidence of the app’s impact on caregiver outcomes. Similar studies have highlighted the importance of rigorous research designs to establish causality. For instance, the study by Sari and colleagues employed an RCT to evaluate the effectiveness of a ‘WANTER’ mobile health intervention, demonstrating its impact on health outcomes more conclusively [23].

Second, while our study primarily focused on quantitative metrics, such as perceived usefulness and ease of use, which provided data on the app’s effectiveness, it lacked in-depth qualitative insights from caregivers regarding their user experience (UX) and challenges with the app. Another study by Zhu and co-workers used an RCT to test how well a digital health intervention worked. Older people could encode, store, and retrieve non-declarative memories of its benefits [24]. Future studies should incorporate qualitative methods, such as focus group discussions and in-depth interviews, to gather comprehensive feedback from caregivers. This feedback will uncover critical areas for improvement and provide a deeper understanding of how the app can be optimized to meet caregivers’ needs effectively. Qualitative insights are essential for understanding the nuances of user experience.

Lastly, we conducted our study in the rural setting of Maha Sarakham province, Thailand, and found significant improvements in caregiver support and resource management. However, its generalizability to other rural or cultural settings with different socio-economic contexts remains a concern. The unique demographic and geographic characteristics of Maha Sarakham may not fully represent other regions. Future research should explore the scalability and adaptability of the “*SmartCaregivers*” mobile application in diverse areas. Additionally, customizing the app to address caregivers’ specific needs and challenges in different cultural and socio-economic contexts could enhance its effectiveness and generalizability. Addressing these aspects will help determine the actual effectiveness of the app in improving care quality and support for long-term dependent individuals.

## 5. Conclusions

The development and evaluation of the “*SmartCaregivers*” mobile application demonstrate its effectiveness in supporting caregivers of long-term dependent individuals in rural areas of Maha Sarakham province. The study underscores the importance of user-centered design and continuous feedback in creating solutions that meet the specific needs of caregivers. Future research should explore the long-term impact of such applications and investigate their scalability to other regions. The positive reception and significant improvements observed in this study suggest that mobile health applications have the potential to transform caregiving practices and improve the quality of life for both caregivers and dependent individuals.

## Figures and Tables

**Table 1:** Socio-economic information and the need to develop a database system for the mobile application (*n* = 402).

Socio-Economic Information	Number (People)	Percentage
Gender		
Male	17	4.2
Female	385	95.8
Age group		
Early Adulthood (18–34 years)	21	5.2
Middle age (35–59 years)	336	83.6
Older Adulthood (60 years and above)	45	11.2
Mean = 49.89 years, SD = 8.14, Max = 75 years, Min = 23 years		
Marital status		
Single	31	7.7
Married	316	78.6
Divorced/Widowed/Separated	55	13.7
Number of Family Members		
1–4 people	192	47.8
5–7 people	193	48.0
8–10 people	17	4.2
Mean = 4.71 people, SD = 1.60, Max = 10 people, Min = 1 person		
Living situation		
Living alone	15	3.7
Living with spouse	273	67.9
Living with children/grandchildren	78	19.4
Living with relatives	17	4.2
Others	19	4.8
Education level		
No formal education	1	0.2
Primary education	61	15.2
Secondary education	294	73.1
Diploma	30	7.5
Bachelor’s degree	14	3.5
Higher than a bachelor’s degree	2	0.5
Average monthly income		
Less than 5000 Baht (153 USD)	239	59.5
5000–10,000 Baht (153–307 USD)	156	38.8
10,001–15,000 Baht (307–406 USD)	3	0.7
More than 15,000 Baht (406 USD)	4	1.0
Mean = 4193.88 Baht (128 USD), SD = 3350.77, Max = 40,000 Baht (1227 USD), Min = 600 Baht (18 USD)		
Sufficiency of Income		
Insufficient and in debt	155	38.6
Barely sufficient	214	53.2
Sufficient	25	6.2
Able to save	52	12.9
Level of need for developing a database system for the mobile application		
Highest (4.21–5.00)	141	35.15
High (3.41–4.20)	243	60.26
Moderate (2.61–3.40)	18	4.59
Low (1.81–2.60)	0	0.00
Lowest (1.00–1.80)	0	0.00
Mean = 4.11, SD = 0.69, Min = 2.96, Max = 5.00		

**Table 2:** Comparison of mean knowledge scores on using the “*SmartCaregivers*” mobile application before and after using the application in the experimental group (*n* = 340).

Caregiver’s Knowledge	*n*	Mean (SD)	Mean Difference	95% Mean Difference		*t*	df	*p*-Value
				Lower	Upper			
Pre-test	340	10.49 (2.53)	−1.69	−2.15	−1.22	−7.12	339	<0.001
Post-test	340	12.18 (2.76)						

**Table 3:** Evaluation of usefulness and ease of use of the “*SmartCaregivers*” mobile application (*n* = 402).

Questions	Mean Score	SD
Perceived usefulness		
(1) “*SmartCaregivers*” helps you receive health information and updates quickly.	4.36 (Highest)	0.62
(2) “*SmartCaregivers*” helps you receive accurate and reliable health information.	4.30 (Highest)	0.53
(3) ”*SmartCaregivers*” is beneficial for making decisions about caring for dependent individuals in the community.	4.30 (Highest)	0.57
(4) “*SmartCaregivers*” can improve work efficiency.	4.26 (Highest)	0.59
(5) “*SmartCaregivers*” helps coordinate between internal and external organizations.	4.26 (Highest)	0.60
Perceived ease of use		
(6) *“SmartCaregivers”* makes caregiving tasks for dependent individuals more convenient and quicker.	4.31 (Highest)	0.59
(7) “S*martCaregivers”* is easily accessible and convenient for mobile devices.	4.26 (Highest)	0.60
(8) *“SmartCaregivers*” improves the accuracy of analyzing the locations of dependent individuals.	4.18 (High)	0.60
(9) *“SmartCaregivers*” makes processing and utilizing data from the health service database easier.	4.16 (High)	0.58
(10) *“SmartCaregivers*” has easy steps for accessing information.	4.11 (High)	0.61
Attitude toward using the application		
(11) *“SmartCaregivers*” can be practically implemented in their work.	4.24 (Highest)	0.57
(12) Caregivers believe *“SmartCaregivers”* is essential for integration with the health service system.	4.19 (High)	0.57
(13) Caregivers have been willing to use “*SmartCaregivers”* in their current tasks.	4.13 (High)	0.58
(14) “*SmartCaregivers*” makes caregivers want to use it again in the future.	4.09 (High)	0.62
(15) Caregivers want to use “*SmartCaregivers*” to develop other work areas.	4.03 (High)	0.64
Behavioral intention to use the application		
(16) Caregivers intend to use “*SmartCaregivers*” to analyze health data to assist in caregiving for dependent individuals.	4.19 (High)	0.61
(17) Caregivers intend to manage the database system used to develop the health service system for dependent individuals.	4.18 (High)	0.60
(18) Caregivers intend to use *“SmartCaregivers*” to assist in planning and implementing work processes to develop ongoing care services for dependent individuals.	4.17 (High)	0.61
(19) Caregivers voluntarily use the “*SmartCaregivers*” to assist in caregiving for dependent individuals under their responsibility.	4.16 (High)	0.64
(20) Using *“SmartCaregivers*” increases their workload.	3.36 (High)	1.08
Actual system use		
(21) Caregivers are confident using “*SmartCaregivers*” to assist in caregiving operations for dependent individuals.	4.11 (High)	0.61
(22) Caregivers use “*SmartCaregivers*” to analyze health situations and plan care for dependent individuals.	3.89 (High)	0.67
(23) Caregivers use *“SmartCaregivers*” to forecast future health trends of dependent individuals.	3.88 (High)	0.71
(24) Caregivers can comprehensively access all menus within the application.	3.70 (High)	0.77
(25) Caregivers are knowledgeable about using various menus in “*SmartCaregivers*”.	3.66 (High)	0.74
System quality		
(26) Caregivers think “*SmartCaregivers*” has various functions.	4.02 (High)	0.59
(27) Caregivers think *“SmartCaregivers*” has an excellent structural system.	4.00 (High)	0.58
(28) Caregivers think “*SmartCaregivers*” has appropriate functions.	3.93 (High)	0.61
(29) Caregivers think “*SmartCaregivers*” is easy to use.	3.85 (High)	0.65

## Data Availability

Data is available upon request.

## Supplementary Materials

The following supporting information can be downloaded at: https://www.mdpi.com/article/10.3390/healthcare12151473/s1, File S1: Questionnaire in English; File S2: Questionnaire in Thai; File S3: Total results, knowledge before/after and questionnaires and codes; File S4: Summary of the Group discussion and In-depth interviews on Capacity Building for Caregivers of Long-term Dependent Individuals in Maha Sarakham Province.
