# Supplementary material for: A Mobile Application for Enhancing Caregiver Support and Resource Management for Long-Term Dependent Individuals in Rural Areas
Source: Healthcare (Basel). 2024 Jul 24;12(15):1473. doi: 10.3390/healthcare12151473 (PMC11311701; doi:10.3390/healthcare12151473)
Supplement: Supplementary file 1 [file healthcare-12-01473-s001.zip › Supplementary data S1.pdf]

The Technology Acceptance Model (TAM) Questionnaires

- -

Instructions: This questionnaire is for the project's target group to develop caregivers' potential in the long-term care system in Maha Sarakham Province. It is divided into 3 phases as follows:

Phase 1 (Analysis): Survey general information on caregivers in the long-term care system and administrators and managers of the public health long-term care system.

Phase 2 and 3 (Design and Development): Assessment of knowledge in elderly healthcare during the trial use of the Smart Caregiver application for the caregiver group.

Phase 4 and 5 (Implementation and Evaluation): Evaluation of acceptance of the application technology for the development of the health database system for caregivers and those with long-term dependence and evaluation of the quality of the application.

The researchers request your cooperation in providing information in this questionnaire. The information obtained from you will be kept confidential and used for research purposes only. The researchers assure you that it will not affect you in any way. Therefore, we kindly request everyone to read or listen to the questions in detail and answer the questions as closely as possible to your opinion. If the evaluator feels uncomfortable answering, they can stop providing information without prior notice to the researcher. If you need more details about the research, you can contact Associate Professor Dr. Niruwan Turnbull, the research project leader, at 086-854-3879.

We want to thank everyone who has sacrificed their time and cooperated to provide information on this occasion.

Associate Professor Dr. Niruwan Turnbull,

Research Project Leader

Phase 1: Survey of General information

Section 1: General Information

1. **User group:**

☐ Caregiver (CG)

☐ Local volunteer caregiver (Care Community: CC)

☐ Long-term care system manager in public health (Care Manager: CM)

☐ Director of public health service unit (District Hospital/Sub-district Health Promotion Hospital/Health Center)

☐ Administrator of local administrative organization (Sub-District Administrative Organization/ Municipal/City Municipality)

2. **Participating network area:**

District: ….. Sub-District: …..

Organization:…………………………...

Section 2 Questionnaire for Caregivers (CG) in the Long-term Care System

Instructions: Please mark the box and fill the blank space with the most truthful information.

**Questions**
1) Gender: ❒ 1) Male ❒ 2) Female
2) Age: …........................ years
3) Marital status: ❒ 1) Single ❒ 2) Married ❒ 3) Divorced/Widowed ❒ 4) Separated
4) Number of family members: …....................... people (including the respondent)
5) Number of children: …................ people
6) Living arrangement: ❒ 1) Living alone ❒ 2) Living with spouse ❒ 3) Living with children/grandchildren ❒ 4) Living with relatives ❒ 5) Other (please specify) …...
7) Education level: ❒ 1) No education ❒ 2) Primary school ❒ 3) Secondary school ❒ 4) Diploma ❒ 5) Bachelor's degree ❒ 6) Higher than bachelor's degree
8) Average monthly income: …................................ Baht
9) Sufficiency of income: ❒ 1) Deficient and in debt ❒ 2) Barely enough some months ❒ 3) Barely enough some months ❒ 4) Able to save
10) Please provide your opinion on the need for the development of a database system for the mobile application. ❒ 1) Strongly agree ❒ 2) Agree ❒ 3) Moderate ❒ 4) Disagree ❒ 5) Strongly disagree

Section 3 Guidelines for group discussion and brainstorming to develop the potential of caregivers using a Smart Caregiver Application are provided for administrators and managers of the public health long-term care system.

1. **What information do caregivers need regarding long-term care?**
   ……………………………………………………………………………………………
   ……………………………………………………………………………………………
   ……………………………………………………………………………………………
   ……………………………………………………………………………………………
   ………………………………………………………………………………………
2. **How can knowledge management be improved?**
   ……………………………………………………………………………………………
   ……………………………………………………………………………………………
   ……………………………………………………………………………………………
   ……………………………………………………………………………………………
   ………………………………………………………………………………………
3. **What are the mapping and tracking needs for caregivers?**
   ……………………………………………………………………………………………
   ……………………………………………………………………………………………
   ……………………………………………………………………………………………
   ……………………………………………………………………………………………
   ………………………………………………………………………………………
4. **How can reporting be streamlined?**
   ……………………………………………………………………………………………
   ……………………………………………………………………………………………
   ……………………………………………………………………………………………
   ……………………………………………………………………………………………
   ………………………………………………………………………………………
5. **What are the biggest problems in caring for people with dependence?**
   ……………………………………………………………………………………………
   ……………………………………………………………………………………………
   ……………………………………………………………………………………………

Phase 2 and 3 Assessment of knowledge on older adult healthcare during the trial use of the SmartCaregiver application by the caregiver group

Instructions: Please select 20 questions that best match your opinion.

Question	Answer	
	True	False
1. A finger prick is one method for testing blood sugar levels.		
2. The normal blood sugar level is 100 mg/L before a meal.		
3. Blood pressure has two numeric values: the top and bottom numbers.		
4. Normal blood pressure is not more than 120/80 mmHg.		
5. Caring for older adults is the same as caring for children.		
6. We use the 2Q and 9Q assessment tools to screen for hypertension.		
7. If older adults have trouble hearing or are incapable of hearing, you should shout to help them hear more clearly.		
8. In old age, energy metabolism decreases, so older adults should receive a variety of foods from all five food groups but in smaller portions than young adults.		
9. If older adults have chronic constipation, the caregiver should give them laxatives daily to prevent abdominal discomfort and bloating.		
10. If you experience joint or muscle pain during exercise, you should stop exercising immediately.		
11. When brushing older adults' teeth, the caregiver should also clean the cheeks, gums, and tongue.		
12. Painting different floor levels in a home with elderly residents in contrasting colors can cause dizziness and increase older adults' risk of falls.		
13. When cleaning older adults' genital area, wipe from bottom to top.		
14. Position older adults with their heads raised to facilitate easier swallowing and prevent choking.		
15. The normal body temperature is 37.6-38.4 degrees Celsius.		
16. The assessment of activities of daily living (ADL) has 90 items.		
17. You can calculate the body mass index (BMI) using your weight and waist circumference.		
18. The principle of giving medication to older adults is the right disease, the right person, the right time, the right method, and the right dosage.		
19. The ADL assessment aids in the screening and appropriate care planning for those with dependence.		
20. Patients receiving tube feeding do not need to practice oral hygiene.		

Phase 4 and 5 Evaluation of the acceptance of the application technology

Evaluation of acceptance of the application technology for the development of the health database system for caregivers and those with long-term dependence (29 items)

Instructions: Please select the option that best matches your opinion.

Question	Level of Opinion				
	Highest (5)	High (4)	Moderate (3)	Low (2)	Lowest (1)
**Perceived Usefulness**					
1. The 'SmartCaregivers' helps you receive health information and updates quickly.					
2. The 'SmartCaregivers' helps you receive accurate and reliable health information					
3. The 'SmartCaregivers' is beneficial for making decisions about caring for dependent individuals in the community.					
4. The 'SmartCaregivers' can improve work efficiency.					
5. The 'SmartCaregivers' helps coordinate between internal and external organizations.					
**Perceived Ease of Use**					
6. The 'SmartCaregivers' makes caregiving tasks for dependent individuals more convenient and quicker.					
7. The 'SmartCaregivers' is easily accessible and convenient for mobile devices.					
8. The 'SmartCaregivers' improves the accuracy of analyzing the locations of dependent individuals.					
9. The 'SmartCaregivers' makes processing and utilizing data from the health service database easier.					
10. The 'SmartCaregivers' has easy steps for accessing information.					
**Attitude Toward Using**					
11. The 'SmartCaregivers' can be practically implemented in their work.					
12. Caregivers believe the 'SmartCaregivers' is essential for integration with the health service system.					
13. Caregivers have been willing to use the 'SmartCaregivers' in their current tasks.					
14. The 'SmartCaregivers' makes caregivers want to use it again in the future.					
15. Caregivers want to use the 'SmartCaregivers' to develop other work areas.					
**Behavior Intention to Use**					
16. Caregivers intend to use the 'SmartCaregivers' to analyze health data to assist in caregiving for dependent individuals.					

Question	Level of Opinion				
	Highest (5)	High (4)	Moderate (3)	Low (2)	Lowest (1)
17. Caregivers intend to manage the database system used to develop the health service system for dependent individuals.					
18. Caregivers intend to use the 'SmartCaregivers' to assist in planning and implementing work processes to develop ongoing care services for dependent individuals.					
19. Caregivers voluntarily use the 'SmartCaregivers' to assist in caregiving for dependent individuals under their responsibility.					
20. Using the 'SmartCaregivers' increases their workload.					
**Actual System Use**					
21. Caregivers are confident using the "SmartCaregivers' to assist in caregiving operations for dependent individuals.					
22. Caregivers use the 'SmartCaregivers' to analyze health situations and plan care for dependent individuals.					
23. Caregivers use the 'SmartCaregivers' to forecast future health trends of dependent individuals.					
24. Caregivers can comprehensively access all menus within the application.					
25. Caregivers are knowledgeable about using various menus in the 'SmartCaregivers'.					
**System quality**					
26. Caregivers think the 'SmartCaregivers' has various functions.					
27. Caregivers think the 'SmartCaregivers' has an excellent structural system.					
28. Caregivers think the 'SmartCaregivers' had appropriate functions.					
29. Caregivers think 'SmartCaregivers' is easy to use.					
