# Supplementary material for: A Mobile Application for Enhancing Caregiver Support and Resource Management for Long-Term Dependent Individuals in Rural Areas
Source: Healthcare (Basel). 2024 Jul 24;12(15):1473. doi: 10.3390/healthcare12151473 (PMC11311701; doi:10.3390/healthcare12151473)
Supplement: Supplementary file 1 [file healthcare-12-01473-s001.zip › Supplementary data S2.pdf]

แบบประเมินการยอมรับเทคโนโลยีสารสนเทศ (Technology Acceptance Model: TAM)

โครงการพัฒนาแอปพลิเคชันระบบฐานข้อมูลสารสนเทศทางภูมิศาสตร์ของกลุ่มผู้ช่วยเหลือดูแล

ผู้ที่มีภาวะพึ่งพิงระยะยาว ในเขตพื้นที่จังหวัดมหาสารคาม (โครงการย่อยที่ 4)

ภายใต้โครงการพัฒนาศักยภาพผู้ช่วยเหลือดูแลผู้ที่มีภาวะพึ่งพิงในระบบการดูแลระยะยาว

คำชี้แจง:

แบบประเมินนี้ใช้สำหรับกลุ่มเป้าหมายของโครงการพัฒนาศักยภาพผู้ช่วยเหลือดูแลผู้ที่มีภาวะพึ่งพิงในระบบการดูแลระยะยาว ในเขตพื้นที่จังหวัดมหาสารคาม แบ่งออกเป็น 4 ส่วน โดยแบ่งเป็นการสอบถาม 3 ระยะ ดังนี้

ระยะที่ 1:

แบบสอบถามการสำรวจข้อมูลทั่วไปของผู้ช่วยเหลือดูแลผู้ที่มีภาวะพึ่งพิงในระบบการดูแลระยะยาว และผู้บริหารและผู้จัดการระบบการดูแลระยะยาวด้านสาธารณสุข

ระยะที่ 2 และ 3: แบบสอบถามเพื่อประเมินความรู้ด้านการดูแลสุขภาพผู้สูงอายุ ในช่วงทดลองใช้แอปพลิเคชันระบบฐานข้อมูลสารสนเทศทางภูมิศาสตร์ของกลุ่มผู้ช่วยเหลือดูแล

ระยะที่ 4 และ 5:

แบบประเมินการยอมรับการใช้เทคโนโลยีแอปพลิเคชันการพัฒนาระบบฐานข้อมูลสุขภาพของผู้ช่วยเหลือดูแลและผู้ที่มีภาวะพึ่งพิงระยะยาว และแบบประเมินเกี่ยวกับคุณภาพของแอปพลิเคชัน

คณะผู้วิจัยขอความร่วมมือในการให้ข้อมูลในแบบประเมินนี้ ซึ่งข้อมูลที่ได้จากท่านจะถือเป็นความลับและนำข้อมูลไปใช้ประโยชน์ในการศึกษาวิจัยเท่านั้น ผู้วิจัยขอให้การรับรองว่าจะไม่มีผลต่อตัวท่านแต่ประการใด จึงขอความอนุเคราะห์จากทุกท่าน ได้อ่านหรือฟังคำถามโดยละเอียด แล้วตอบคำถามให้ตรงกับความคิดเห็นของท่านมากที่สุด ถ้าผู้ประเมินมีความอึดอัดใจในการตอบ สามารถขอหยุดการให้ข้อมูลได้ โดยไม่จำเป็นต้องแจ้งให้ผู้วิจัยทราบล่วงหน้า และถ้าต้องการรายละเอียดของการวิจัยสามารถติดต่อ สอบถามได้ที่ รองศาสตราจารย์ ดร.นิรุวรรณ เทิร์นโบล์ หัวหน้าโครงการวิจัย หมายเลขโทรศัพท์ 086-854-3879

ขอขอบพระท่านทุกท่าน ที่เสียสละเวลาและให้ความร่วมมือในการให้ข้อมูลในครั้งนี้

ระยะที่ 1

การสำรวจข้อมูลทั่วไปของผู้ช่วยเหลือดูแลผู้ที่มีภาวะพึ่งพิงในระบบการดูแลระยะยาว และผู้บริหารและผู้จัดการระบบการดูแลระยะยาวด้านสาธารณสุข

ส่วนที่ 1 ข้อมูลทั่วไป

คำชี้แจง: โปรดให้ข้อมูลที่ตรงกับความเป็นจริงกับตัวท่านมากที่สุด

1.กลุ่มผู้ใช้งาน

- ☐ ผู้ช่วยเหลือดูแล (Care Giver: CG)
- ☐ อาสาสมัครบริบาลท้องถิ่น (Care Community: CC)
- ☐ ผู้จัดการระบบการดูแลระยะยาวด้านสาธารณสุข (Care Manager: CM)
- ☐ ผู้อำนวยการหน่วยบริการสาธารณสุข (รพช./รพ.สต./สอน.)
- ☐ ผู้บริหารองค์กรปกครองส่วนท้องถิ่น (อบต./ทต./ทม.)

2.พื้นที่ร่วมเครือข่าย

อำเภอ .... ตำบล ....

หน่วยงาน ....

<u>ส่วนที่ 2</u> แบบสอบถามสำหรับผู้ช่วยเหลือดูแลผู้ที่มีภาวะพึ่งพิงในระบบการดูแลระยะยาว (CG)

คำชี้แจง: โปรดทำเครื่องหมาย ✓ลงในช่อง ❒ และเติมข้อความลงในช่องว่างให้ตรงกับความเป็นจริงที่สุด

ข้อคำถาม
1) เพศ<br>❒ 1) ชาย ❒ 2) หญิง
2) อายุ .... ปี
3) สถานภาพ ❒ 1) โสด ❒ 2) สมรส<br>❒ 3) หย่า / หม้าย ❒ 4) แยกกันอยู่
4) จำนวนสมาชิกในครอบครัว .... คน (รวมผู้ตอบด้วย)
5) จำนวนบุตร .... คน
6) ลักษณะการอยู่อาศัย<br>❒ 1) อยู่ตามลำพัง ❒ 2) อยู่กับคู่สมรส<br>❒ 3) อยู่กับบุตร/หลาน ❒ 4) อยู่กับญาติ<br>❒ 5) อื่น ๆ (โปรดระบุ) ....
7) ระดับการศึกษา<br>❒ 1) ไม่ได้เรียน ❒ 2) ประถมศึกษา<br>❒ 3) มัธยมศึกษา ❒ 4) อนุปริญญา<br>❒ 5) ปริญญาตรี ❒ 6) สูงกว่าปริญญาตรี
8) รายได้เฉลี่ย .... บาทต่อเดือน
9) ความเพียงพอของรายได้<br>❒ 1) ขัดสนและมีหนี้สิน ❒ 2) พอใช้บางเดือน<br>❒ 3) พอใช้อยู่ได้สบาย ๆ ❒ 4) เหลือเก็บออม
10) โปรดให้ความเห็นต่อความจำเป็นในการมีแอปมือถือสำหรับการจัดการข้อมูล<br>❒ 1) น้อยที่สุด ❒ 2) น้อย ❒ 3) ปานกลาง ❒ 4) มาก ❒ 5) มากที่สุด

ส่วนที่ 3 แนวทางการสนทนากลุ่ม

1. คุณต้องการมีข้อมูลประเภทใดเกี่ยวกับการดูแลระยะยาว (LTC) ที่คุณดูแล

   ………

   ………

   ………

   ………

   ………………………………

2. คุณจะจัดการความรู้ของคุณอย่างไร? (Knowledge Management: KM)

   ………

   ………

   ………

   ………

   ………………………………

3. คุณจะจัดระเบียบการจัดการแผนการดูแลสำหรับผู้ดูแลอย่างไร (CG)?

   ………

   ………

   ………

   ………

   ………………………………

4. ในระบบ CG คุณจะจัดระเบียบการเขียนรายงานผลการปฏิบัติงานอย่างไร?

   ………

   ………

   ………

   ………

   ………………………………

5. ปัญหาที่ใหญ่ที่สุดในการดูแลผู้พึ่งพาอาศัยกันคืออะไร?

   ………

   ………

   ………

   ……………………………………………………

ระยะ 2 และ 3 การสอบถามเพื่อประเมินความรู้ด้านการดูแลสุขภาพผู้สูงอายุ

ในช่วงทดลองใช้แอปพลิเคชันระบบฐานข้อมูลสารสนเทศทางภูมิศาสตร์ของกลุ่มผู้ช่วยเหลือดูแล

คำชี้แจง: โปรดเลือกตัวเลือกที่ท่านเห็นว่าถูกต้องที่สุด

คำถาม	คำตอบ	
	ใช่	ไม่ใช่
1. การตรวจระดับน้ำตาลในเลือด มี 1 วิธี คือการเจาะที่ปลายนิ้ว		
2. ค่าระดับน้ำตาลปกติก่อนรับประทานอาหาร คือ 100 mg/L		
3. ค่าของความดันโลหิตจะมีลักษณะตัวเลข 2 คือ ค่าความดันตัวบน และค่าความดันตัวล่าง		
4. ระดับความดันโลหิตปกติ คือไม่เกิน 120/80 มม.ปรอท		
5. การดูแลผู้สูงอายุเหมือนกับการดูแลเด็ก		
6.แบบประเมิน 2Q และ 9Q ใช้คัดกรองโรคความดันโลหิตสูง		
7. ถ้าผู้สูงอายุได้ยินไม่ชัดเจน หรือหูตึง ควรตะโกนพูดเสียงดังเพื่อช่วยให้ผู้สูงอายุได้ยินชัดเจนขึ้น		
8. วัยสูงอายุ การเผาผลาญพลังงานลดลง ควรได้รับอาหารให้หลากหลายชนิดครบทั้ง 5 หมู่ แต่ปริมาณควรลดน้อยลงกว่าวัยหนุ่มสาว		
9. หากผู้สูงอายุท้องผูกเป็นประจำผู้ดูแลควรให้ยาระบายทุกวันเพื่อไม่ให้รู้สึกแน่นอึดอัดท้อง		
10. ขณะออกกำลังกายถ้ามีอาการปวดตามข้อต่างๆ หรือกล้ามเนื้อ ควรหยุดออกกำลังกายทันที		
11. การแปรงฟันให้ผู้สูงอายุควรต้องทำความสะอาดบริเวณกระพุ้งแก้ม เหงือก และลิ้นด้วย		
12. บ้านที่มีผู้สูงอายุอยู่ด้วย ถ้ามีพื้นต่างระดับ ไม่ควรทาสีแตกต่างกัน เพราะจำทำให้ผู้สูงอายุเวียนศีรษะเกิดอุบัติเหตุ ล้มได้ง่าย		
13. การทำความสะอาดบริเวณอวัยวะขับถ่ายให้แก่ผู้สูงอายุควรเช็ดจากด้านล่างขึ้นด้านบน		
14. การป้อนอาหารควรจัดให้ผู้สูงอายุนอนศีรษะสูง เพื่อกลืนอาหารสะดวกและป้องกันการสำลักอาหาร		
15. อุณหภูมิของร่างกายปกติ คือ 37.6-38.4 องศาเซลเซียส		
16. การประเมินความสามารถในการทำกิจวัตรประจำวัน (ADL) มีทั้งหมด 90 ข้อ		
17.ค่าดัชนีมวลกาย (Body Mass Index: BMI) สามารถคำนวณได้จากค่าน้ำหนักและเส้นรอบเอว		
18. หลักในการให้ยาแก่ผู้สูงอายุ คือ ถูกโรค ถูกคน ถูกเวลา ถูกวิธี และถูกขนาด		
19. การประเมินค่า ADL ช่วยในการคัดแยกและวางแผนดูแลผู้ที่มีภาวะพึ่งพิงให้เหมาะสม		
20. ไม่จำเป็นต้องทำความสะอาดช่องปากของผู้ป่วยที่ได้รับอาหารทางสายยาง		

ระยะที่ 4 และ 5 การประเมินการยอมรับการใช้เทคโนโลยีแอบพลิเคชัน

ระบบฐานข้อมูลสุขภาพของผู้ช่วยเหลือดูแลและผู้ที่มีภาวะพึ่งพิงระยะยาว (จำนวน 29 ข้อ)

คำชี้แจง: โปรดเลือกตัวเลือกที่ตรงกับความคิดเห็นของท่านมากที่สุด

**คำถาม**	**ระดับความคิดเห็น**				
	**มากที่สุด (5)**	**มาก (4)**	**ปานกลาง (3)**	**น้อย (2)**	**น้อยที่สุด (1)**
**การรับรู้ว่าแอปพลิเคชันมีประโยชน์ (Perceived Usefulness)**					
1.แอปพลิเคชัน "SMART Caregiver" ช่วยให้ท่านได้รับข้อมูลและข่าวสารด้านสุขภาพได้อย่างรวดเร็ว					
2.แอปพลิเคชัน "SMART Caregiver" ช่วยให้ท่านได้รับข้อมูลและข่าวสารด้านสุขภาพที่ถูกต้องและเชื่อถือได้					
3.แอปพลิเคชัน "SMART Caregiver" มีประโยชน์ต่อการตัดสินใจวางแผนการช่วยเหลือดูแลผู้ที่มีภาวะพึ่งพิงและผู้สูงอายุในชุมชนได้					
4.แอปพลิเคชัน "SMART Caregiver" สามารถเพิ่มประสิทธิภาพในการปฏิบัติงาน					
5.แอปพลิเคชัน "SMART Caregiver" ช่วยในการประสานงานระหว่างองค์กรทั้งภายในและภายนอก					
**การประเมินความยากง่ายในการใช้แอปพลิเคชัน (Perceived Ease of Use)**					
6.แอปพลิเคชัน "SMART Caregiver" มีขั้นตอนที่ง่ายต่อการเข้าถึงข้อมูล					
7.แอปพลิเคชัน "SMART Caregiver" ช่วยให้การทำงานด้านการดูแลสุขภาพผู้ที่มีภาวะพึ่งพิงสะดวกและรวดเร็วขึ้น					

คำถาม	ระดับความคิดเห็น				
	มากที่สุด (5)	มาก (4)	ปานกลาง (3)	น้อย (2)	น้อยที่สุด (1)
8.แอปพลิเคชัน "SMART Caregiver" ช่วยลดขั้นตอนการทำงานให้น้อยลง					
9.แอปพลิเคชัน "SMART Caregiver" สามารถประมวลผลจากระบบฐานข้อมูลในระบบบริการสุขภาพมาใช้ประโยชน์ได้ง่ายขึ้น					
10.แอปพลิเคชัน "SMART Caregiver" สามารถเข้าถึงได้ง่ายสะดวกในการใช้งานในระบบโทรศัพท์มือถือ					
**ด้านทัศนคติและความสนใจที่จะใช้แอปพลิเคชัน (Attitude Toward Using)**					
11.ท่านมีความสนใจที่จะนำแอปพลิเคชัน "SMART Caregiver" ไปใช้ในการพัฒนางานด้านอื่น ๆ					
12.แอปพลิเคชัน "SMART Caregiver" ทำให้ท่านรู้สึกอยากใช้งานในครั้งต่อไปอีก					
13.แอปพลิเคชัน "SMART Caregiver" สามารถนำไปใช้ในการปฏิบัติงานได้จริง					
14.ท่านคิดว่าแอปพลิเคชัน "SMART Caregiver" มีความสำคัญในการประยุกต์ใช้กับระบบบริการสุขภาพ					
15.ท่านมีความคิดที่จะนำแอปพลิเคชัน "SMART Caregiver" ไปใช้ในงานที่ท่านปฏิบัติอยู่					
**ด้านพฤติกรรมในความสนใจที่จะใช้แอปพลิเคชัน (Behavior Intention to Use)**					
16.ท่านมีความตั้งใจที่จะนำแอปพลิเคชัน "SMART Caregiver" มาช่วยในการวางแผนปฏิบัติงานเพื่อพัฒนาการดำเนินงานด้านการช่วยเหลือดูแลผู้ที่มีภาวะพึ่งพิงต่อไป					
17.ท่านมีความตั้งใจที่จะใช้แอปพลิเคชัน "SMART Caregiver" มาช่วยในการวิเคราะห์ข้อมูลทางด้านสุขภาพในการช่วยเหลือดูแลผู้ที่มีภาวะพึ่งพิง					
18.ท่านมีความตั้งใจที่จะใช้แอปพลิเคชัน "SMART Caregiver" มาใช้ในการจัดการระบบฐานข้อมูลเพื่อการพัฒนาระบบบริการสุขภาพของผู้ที่มีภาวะพึ่งพิง					

คำถาม	ระดับความคิดเห็น				
	มากที่สุด (5)	มาก (4)	ปานกลาง (3)	น้อย (2)	น้อยที่สุด (1)
19.ท่านใช้แอปพลิเคชัน "SMART Caregiver" ในการดำเนินงานช่วยเหลือดูแลผู้ที่มีภาวะพึ่งพิงที่อยู่ในความรับผิดชอบของท่านด้วยความสมัครใจ					
20.การใช้แอปพลิเคชัน "SMART Caregiver" เป็นการเพิ่มภาระงานของท่าน					
**ด้านการยอมรับแอปพลิเคชัน (Actual System Use)**					
21.ท่านมีความมั่นใจที่จะนำแอปพลิเคชัน "SMART Caregiver" มาใช้ในการดำเนินงานช่วยเหลือดูแลผู้ที่มีภาวะพึ่งพิง					
22.ท่านมีความรู้ในการใช้เมนูต่าง ๆ ในแอปพลิเคชัน "SMART Caregiver"					
23.ท่านสามารถเข้าถึงระบบแอปพลิเคชัน "SMART Caregiver" ได้อย่างครอบคลุมทุกเมนู					
24.ท่านได้นำ แอปพลิเคชัน "SMART Caregiver" ไปใช้ในการวิเคราะห์สถานการณ์ด้านสุขภาพเพื่อการวางแผนการดูแลผู้ที่มีภาวะพึ่งพิง					
25.ท่านได้นำ แอปพลิเคชัน "SMART Caregiver" ไปใช้ในการพยากรณ์แนวโน้มสถานการณ์ด้านสุขภาพของผู้ที่มีภาวะพึ่งพิงในอนาคต					
**ด้านคุณภาพระบบโครงสร้าง (System Quality)**					
26.ท่านคิดว่า แอปพลิเคชัน "SMART Caregiver" มีฟังก์ชันการทำงานที่เหมาะสม					
27.ท่านคิดว่า แอปพลิเคชัน "SMART Caregiver" ใช้งานง่าย					
28.ท่านคิดว่า แอปพลิเคชัน "SMART Caregiver" มีระบบโครงสร้างการใช้งานที่ดี					
29.ท่านคิดว่า แอปพลิเคชัน "SMART Caregiver" มีฟังก์ชันการทำงานที่หลากหลาย					
