# Supplementary material for: A Mobile Application for Enhancing Caregiver Support and Resource Management for Long-Term Dependent Individuals in Rural Areas
Source: Healthcare (Basel). 2024 Jul 24;12(15):1473. doi: 10.3390/healthcare12151473 (PMC11311701; doi:10.3390/healthcare12151473)
Supplement: Supplementary file 1 [file healthcare-12-01473-s001.zip › Supplementary data S4.pdf]

Summary of the Group discussion and In-depth interviews on Capacity Building for Caregivers of Long-term Dependent Individuals in Maha Sarakham Province

Results of the Brainstorming Session on the Project to Develop the Capacity of Caregivers for Long-term Dependent Individuals

December 3, 2020, Faculty of Public Health

Representatives from Health Service Units (5 people):

- 4 Care Managers (CM) from hospitals/health promotion hospitals:
  - Care Manager from Kosum Phisai Hospital
  - Care Manager from Kae Dam Hospital
  - Care Manager from Nong Saeng Health Promotion Hospital
  - Care Manager from Chuen Chom Health Promotion Hospital
- 1 Director from a Health Promotion Hospital:
  - Director of Non Piban Health Promotion Hospital

Representatives from Local Government Organizations (5 people):

- 1 Head of the Health and Environmental Division:
  - Head of Health and Environmental Division from Maha Sarakham Municipality
- 2 Care Managers from local administrative organizations:
  - Care Manager from Kae Dam Subdistrict Municipality
  - Care Manager from Chiang Yuen Subdistrict Municipality
- 2 Presidents of Subdistrict Administrative Organizations/Municipalities:
  - President of Wang Saeng Subdistrict Administrative Organization
  - President of Na Chueak Subdistrict Municipality

The brainstorming session highlighted issues, obstacles, and relevant data sets regarding the health and well-being of caregivers of long-term dependent individuals.

Questions and Summary of Related Issues/Data Sets for Operations:

1. **What kind of information would you like to have about long-term care (LTC) that you care for?**

- *Knowledge Management (KM):*

- We currently communicate with the Line Application, a mobile application that shares important knowledge.
- Urgent symptom management during emergencies
- Patient groups are changing.
- The data collection form should have a clear format.
- Records of CG (Caregiver) capacity development and continuous training are available.
- Relevant professional knowledge
- There are challenges in providing home care for elderly and diabetic patients, including inadequate family care, substance abuse, insufficient funds for diapers, and specific needs for the elderly with disabilities.

2. How would you manage your knowledge? (Knowledge Management: KM)

- Monthly unification of responsibility zones for caregivers is necessary.
- Check-in using the Thai Chana application: potential conflicts during check-in and check-out if patients' homes are far away; acceptable duration of home visits (e.g., less than 2 hours)

3. How would you organize care plan management for caregivers (CG)?

- Financial documents for department heads
- I suggest creating a monthly application submission instead of each visit, due to the excessive paperwork involved.
- CM assigns daily care data and CG encounters problems.
- The expenses for each elderly visit and the remaining funds are included.
- Home visit records, including BP, symptoms, first aid, and images
- Monthly performance reports

4. In the CG system, how would you organize writing performance reports?

- ***CM perspectives:***

- A clear format and financial system
- The documentation is excessive; it would be beneficial to suggest a work schedule for the CGs.
- Linking CM and CG plans
- System redundancies cause issues.
- Internet recharge issues affecting application usage; suggest offline functionality
- Printable application documents for simple submission to the local administrative organization

- Due to personal constraints, CG work schedules may not align with planned schedules.
- Training is a necessity for CGs with limited application proficiency.
- Additional training courses to alleviate the CM workload

5. What are the biggest problems in caring for people with dependence?

- The monthly work plan
- Issues in caring for dependent individuals:
- Patient information and addresses are lacking.
- Inadequate equipment (budget problems)
- Frequent changes in patient groups
- There is a need for more comprehensive patient data, including medical history and treatment guidelines.
- Tracking budget usage per case
- Database of CGs including training history, personal and family information, and income
